# Dr. Liston on a Variety of False Aneurism

**Published:** 1842-05-14

**Authors:** R. Liston

**Affiliations:** Surgeon to University College Hospital


[The following abstract of a case and comments by Mr. Liston, condensed from a paper read before the Royal Medical and Chirurgical Society, is interesting from the difficulty of diagnosis between aneurism and scrofulous tumor in this instance; but we regret that the details of the appearances on dissection are omitted by the Editor of the Gazette; for it is certainly difficult to understand, as Dr. Johnson pertinently remarked during the debate upon the paper, what could have prevented the appearance of pus, if the aneurism were really a result of intercommunication between the artery and a scrofulous abscess. Mr. Liston, in reply to this objection, stated "that pus might probably have been discerned by the microscope," but if present at all, it might be expected in large quantities; for it cannot be supposed that the secretion of the abscess escaped by being mingled with the circulating fluid; nor could it be absorbed from the churning contents of a false aneurism.]

*On a Variety of False Aneurism.* By R. Liston, Esq. F. R. S. Surgeon to University College Hospital.—The author's attention has lately been particularly directed to the subject of the communication between large blood-vessels and the cysts of abscesses, in consequence of having lately met with a remarkable case, in which the carotid artery opened into a large abscess in the neck.

G. A., æt. 9, suffered from severe illness about six years ago, by which he was left in a very reduced state. Two months back, he had a violent cough, accompanied with fever, and at this time *a small swelling* was observed in the neck, immediately below the right ear. This swelling increased slowly until within three or four days of his reception into the North London Hospital, on the 20th of October, when its progress had become rapid and irregular. At the date mentioned, there was observed a tumor extending from the angle of the jaw on the right side, downwards to within an inch of the clavicle, backwards to the posterior edge of the sterno-mastoid muscle which it raised, and forwards to about midway between the angle of the jaw and chin. The tumor farther projected inwards into the mouth between the arches of the palate, and materially impeded both deglutition and respiration. Indistinct fluctuation could be distinctly felt in the tumor, and there was slight pulsation in it, immediately over the course of the carotid artery; but on grasping the tumor, and examining it from the mouth, no pulsation could be felt. A small puncture was made into the tumor under the impression that it contained matter, but a gush of arterial blood followed the bistoury, and about four ounces were lost in a few seconds. The puncture was readily closed by hare-lip pins and twisted sutures, and the bleeding checked. I resolved on tying the carotid artery next day.

No hæmorrhage occurred in the course of the ensuing night, but the tumor was tense, and had been covered with a cold lotion. On proceeding to the operation, an incision, about an inch and a half in length, was made transversely over the sternal end of the clavicle, and another upwards and at right angles to the first, over and in line of the trachea; by which an angular flap was formed and turned upwards and outwards. The sternal attachment of the sterno-mastoideus being exposed was cut through; the sterno-hyoideus and sterno-thyroideus were next exposed after some dissection, and divided; at length the carotid artery was exposed a little above its origin from the innominata, and tied. The whole difficulty of the operation arose from the necessary smallness of the external incision. The tumor projected so low down into the neck, that it was impossible to procure space by extending the incision upwards, and the artery which lay at a great distance from the surface had to be sought for at the bottom of a small hole. The flap was laid down, and retained by some isinglass plaster. The boy complained very little after the operation, the swelling became smaller and firmer, and the movements of the jaw, which before were much restricted, were now more free and less painful. The pupil of the right eye, too, which had been contracted and only partially sensible to light, was now restored to its proper functions. The patient slept soundly through the night following the operation.

The pins and twisted sutures were removed on the 25th, and strips of isinglass plaster applied in stead. On the 28th, some grumous blood escaped from the opening in the tumor. The patient was cheerful and happy. Things went on prosperously; the tumor sinking in size, till the afternoon of November 3d, when a sudden gust of arterial blood took place from the wound in the fore part of the neck, the ligature being still firm. The hæmorrhage was arrested for the moment by plugging the wound with lint, but a considerable quantity of blood was lost. Hæmorrhage returned six times after this, and the patient finally sank into a state of collapse, 48 hours after the first occurrence of the bleeding.

On examination, the ligature was found to have been close to the origin of the carotid from the innominata. It was not completely separated; a small portion of the external side of the artery still remaining entire. There had been no attempt at the formation of a clot, or if any had formed it must have been expressed with the blood.

The appearance of the tumor, both externally and internally, are most minutely described by the author, as are also the relation of the vessels to the cyst, and the condition of the opening of communication between the carotid artery and the latter. It would be impossible to do justice to these details in the space of a short abstract; suffice it to say, that the author feels himself warranted in deducing from the examination of the parts the conclusion, that the disease was originally a chronic abscess of a scrofulous character, and that the opening into the artery was consequent upon ulceration from without.

The preparation of the parts, together with two drawings made from them in a recent state, were exhibited to the meeting.

The author relates, in confirmation of his views, the details of three other cases, derived from the practice of himself and others, in which large arteries in the neighbourhood of abscesses were opened by ulceration. Our space will not allow us to refer to these in detail.—*London Med. Gaz.* March 18th, 1842.

